# Facile synthesis of Ag@Fe_3_O_4_/ZnO nanomaterial for label-free electrochemical detection of methemoglobin in anemic patients

**DOI:** 10.1038/s41598-023-35737-w

**Published:** 2023-05-29

**Authors:** Ayub Alam, Batool Fatima, Sameera Shafi, Zohaib Sarwar, Dilshad Hussain, Shan E Zahra Jawad, Saadat Majeed, Muhammad Imran, Muhammad Najam-ul-Haq

**Affiliations:** 1 Department of Chemistry, The Islamia University of Bahawalpur, Bahawalpur, Pakistan; 2 Department of Biochemistry, Bahauddin Zakariya University, Multan, 60800 Pakistan; 3 Center for Refining & Advanced Chemicals, King Fahd University of Petroleum & Minerals, Dhahran, 31261 Saudi Arabia; 4 HEJ Research Institute of Chemistry, International Center for Chemical and Biological Sciences, University of Karachi, Karachi, 75270 Pakistan; 5 Institute of Chemical Sciences, Bahauddin Zakariya University, Multan, 60800 Pakistan; 6 Department of Biochemistry, University of Peshawar, Peshawar, Pakistan

**Keywords:** Biochemistry, Chemistry

## Abstract

Methemoglobinemia (MetHb, Fe^3+^) is a chronic disease arising from the unequal distribution of oxyhemoglobin (HbFe^2+^, OHb) in the blood circulatory system. The oxidation of standard oxyhemoglobin forms methemoglobin, causing cyanosis (skin bluish staining). Methemoglobin cannot bind the pulmonary gaseous ligands such as oxygen (O_2_) and carbon monoxide (CO). As an oxidizing agent, the biochemical approach (MetHb, Fe^3+^) is modified in vitro by sodium nitrite (NaNO_2_). The silver-doped iron zinc oxide (Ag@Fe_3_O_4_/ZnO) is hydrothermally synthesized and characterized by analytical and spectroscopic techniques for the electrochemical sensing of methemoglobin via cyclic voltammetry (CV). Detection parameters such as concentration, pH, scan rate, electrochemical active surface area (ECSA), and electrochemical impedance spectroscopy (EIS) are optimized. The linear limit of detection for Ag@Fe_3_O_4_/ZnO is 0.17 µM. The stability is determined by 100 cycles of CV and chronoamperometry for 40 h. The serum samples of anemia patients with different hemoglobin levels (Hb) are analyzed using Ag@Fe_3_O_4_/ZnO modified biosensor. The sensor's stability, selectivity, and response suggest its use in methemoglobinemia monitoring.

## Introduction

Methemoglobinemia is a long-lasting metabolic, idiopathic, genetic, and self-disrupting disorder of oxygen-carrying hemoglobin in the bloodstream of vertebrates^1,2^. Biochemically, oxyhemoglobin (OHb) is a tetrahedral complex having iron [Fe^2+^] as the central metal atom (CMA) and four pyrrole rings attached to (CMA)^3^. The transportation of pulmonary gases (O_2_ and CO) during the metabolic and respiratory cycle is controlled by the iron-containing non-proteinaceous, “heme and prosthetic” group of hemoglobin^4^. Mutations^5^ in α and β chains of globular (OHb) cause diseases such as diabetes (HbA1c)^6^, sickle cell anemia, leukemia, and thalassemia. Congenitally toxic medication, deficiency of cytochrome-*b*_*5,*_ and self-disruption of (OHb) oxidize [Fe^2+^] “prosthetic or heme” to [Fe^3+^] central metal atom^7,8^. The oxidation of ferrous to ferric alters the normal oxyhemoglobin (HbFe^2+^) as methemoglobin (HbFe^3+^) or ferrihemoglobin^9,10^. This variation leads to chronic methemoglobinemia. Methemoglobin causes the non-binding capability of O_2_ with [Fe^3+^], which further leads to the lack of working power of muscles and tissues in vertebrates.

The 8-hydroxy quinone (8-HCQ) in-take causes methemoglobinemia and hemolysis in patients with a deficiency of the (G6DP) enzyme^11^. High and mild (1–5%) levels of (MetHb) lead to the formation of Ar-Amines and Ar-Halides, which cause deficiency of biochemical enzymes (glucose 6-d-phosphate and reductase) and greater concentrations of nitrites and nitrates^1^. The oxyhemoglobin dissociation curve indicates a higher concentration of allosteric ferric [Fe^3+^] ions. The induced shift in hemoglobin increases congenital methemoglobinemia through erythrocytes in anemic patients, termed erythrocytosis^12^. Cyanosis^13^ (skin bluish staining) establishes rapidly in patients with high levels of MetHb (10–15%) than with normal hemoglobin due to oxygen deficiency^2^. The clinical trials find that MetHb levels vary from 10 to 70%. An increase in [Fe^3+^] concentration changes red blood cell (RBCs) color from red to brownish mud, indicating severe cyanosis and hypoxia^14^. Methylene blue (MB) is the first antidote cofactor for NADH reductase, which lowers chronological methemoglobinemia in respiratory and cardiac patients^15^.

Recent studies show that methemoglobin (MetHb) can be synthesized by a controlled reaction of hemoglobin (Hb) with potassium ferrocyanide [K_3_Fe(CN)_6_] at specific temperatures and pressure. Redox reactions convert standard hemoglobin to methemoglobin. Hydrogen peroxide (H_2_O_2_), hydrogen sulfide^16^ (H_2_S), and sodium nitrite (NaNO_2_) are the strong oxidizing agents catalyzing this reaction^17^. Several methods have been adopted for detecting hemoglobin and its components. Spectroscopic and separation techniques such as infrared (IR) spectroscopy, mass spectrometry (MS), fluorometry^18^, fluorescence spectroscopy, gas chromatography^19^ (GC), and high-performance liquid chromatography (HPLC) are used for the hemoglobin detection. Specific gravity, colorimetry, electrochemical techniques, and Kurt electric resistance^20^ are also employed for quantitative and qualitative analysis of methemoglobin.

The developments in electronic, wearable, and robotic technologies have brought variations in mechanical sensors^21^. Electrochemical sensors are inexpensive, durable, sensitive, simple, and portable^22,23^. They have specific mechanical stress/strain properties for biomolecule detection^24^. Advancements in electrical detections and fabricated pathways have a role in developing chemical and electrical composites as electrochemical sensors^25,26^. Electrochemical methods include cyclic voltammetry (CV), electrochemical impedance spectroscopy (EIS)^27^, chronoamperometry (CA), and differential pulse voltammetry (DPV)^28–30^.

Various electrochemical biosensors have been developed for hemoglobin detection. Voltammetric MXene-based two-dimensional (2D) transition metal carbides^31^, tin oxide (SnO_2_) nanoparticles^32^, graphite carbon nitride (G-C_3_N_4_),^33^ platinum (Pt) doped iron phosphorus carbide (FeP-C)^34^, tellurium nanowires doped graphene oxide (TeNWs/GO) nanocomposite^35^, ticlopidine/titanium dioxide (Tic–TiO_2_) nanoparticles^36^, boron-doped grapheme (B-GQDs) quantum dots^37^, and chiral nano-imprinted (Fe_3_O_4_/SiO_2_) polymers^38^ have been reported for the quantitative analysis of hemoglobin. MXene compositional variability, hydrophilicity, elevated metallic conductivities, and large surface make them efficient tools for analyte detection. Although, it faces some challenges during its synthetic process, i.e., no proper termination step and development of new etching layers. A less toxic and eco-friendly method must be introduced for MXene synthesis^31^. SnO_2_ is utilized in different fields, including lithium-ion batteries and dye-sensitized solar cells, due to their high chemical stability and catalytic activity^32^. G-C_3_N_4_ has been extensively employed as an electrochemical chemosensor and a water-splitting agent. Although it is a major limitation, it cannot be utilized alone because of its low conductivity. It is used with semiconductors, metal nanoparticles, carbon material, and metal ions^33^.

In this study, Ag@Fe_3_O_4_/ZnO (SIZO) nanomaterial is synthesized hydrothermally for the quantitative electrochemical sensing of methemoglobin (MetHb) in anemic patients. Methemoglobinemia (anemic) patients carry lower oxygen levels in a metabolic cycle, which leads to cyanosis. The (SIZO) modified electrode shows enhanced electro-catalytic activity and fast electron transfer for in vitro and in vivo electrochemical sensing of methemoglobin compared to other nanomaterials. By far, sensors have been reported to detect hemoglobin, and its link to anemia has been a concern. According to our literature survey, the relationship between sensors for detecting methemoglobin and anemia has never been reported. The fabricated nanocomposite will assist in detecting methemoglobin, making an earlier diagnosis of methemoglobinemia, further leading to cyanosis much easier. The electrochemical analysis of methemoglobin is accomplished in the blood samples of anemic patients. The novel silver-doped iron zinc oxide (SIZO) nanomaterial is being reported for the first time in the electrochemical sensing of methemoglobin (MetHb).

## Experimental section

### Chemicals and reagents

Silver nitrate (AgNO_3_) ≥ 99%, Iron (II) sulfate heptahydrate ≥ 99% (FeSO_4_·7H_2_O), Iron (III) chloride 97% (FeCl_3_), Zinc acetate dehydrate 99.9% Zn(CH_3_CO_2_)_2_·2H_2_O, Standard Hemoglobin (Hb) powder (≥ 99.9%), Sodium nitrite (NaNO_2_) ≥ 99%, absolute ethanol ≥ 99.8% (CH_3_CH_2_OH), Sodium hydroxide 99.99% (NaOH), Ammonium hydroxide (NH_4_OH) 30% solution, Monopotassium dihydrate phosphate 98% (KH_2_PO_4_), Dipotassium monohydrate phosphate 98% (K_2_HPO_4_), Trisodium citrate 99% (C_6_H_5_O_7_Na_3_), and Citric acid 99.99% (C_6_H_8_O_7_) were purchased from Sigma Aldrich USA.

### Synthesis of silver nanoparticles

Silver nanoparticles (Ag-NPs) were prepared by hydrothermal method. Precursor mixtures were prepared in distilled water. 80 mL solution of 0.5 g AgNO_3_ was heated at 60 °C and added to preheated 20 mL solution of (C_6_H_5_O_7_Na_3_) and (C_6_H_8_O_7_) at 60 °C and stirred for 20 min^39^. The mixture was transferred to a 100 mL TEFLON-lined hydrothermal autoclave and placed at 160 °C for 12 h (Eq. 1). The reaction products were washed and purified by deionized water under ultracentrifugation. The washed nanomaterial was dried at 65 °C for 3 days and stored for further use.

1 $$4{\text{Ag}}^{ + } + {\text{C}}_{6} {\text{H}}_{5} {\text{O}}_{7} {\text{Na}}_{3} + 2{\text{H}}_{2} {\text{O}}\mathop{\longrightarrow}\limits_{{160\;^{ \circ } {\text{C}}}}^{{{\text{Hydrothermal}}}}4{\text{Ag}}^{0} + {\text{C}}_{6} {\text{H}}_{5} {\text{O}}_{7} {\text{H}}_{3} + 3{\text{Na}}^{ + } + {\text{H}}^{ + } + {\text{O}}^{ - }$$

### Synthesis of Fe_3_O_4_ nanoparticles

Fe_3_O_4_ NPs were synthesized through the hydrothermal method. The reaction mixture of 0.5 g FeSO_4_·7H_2_O and 0.05 g FeCl_3_ were dissolved in 60 mL distilled water at a 2:1 M ratio and stirred at 40 °C for 4 h, followed by the dropwise addition of 3 mL NH_4_OH. The mixture was autoclaved in a sealed pressure vessel of 55 mL volume at 140 °C for 4 h. The black oxidized precipitates were collected and washed with deionized water and absolute ethanol via ultracentrifugation-dispersion. Fe_3_O_4_ NPs were dried at 60 °C for one week and annealed at 450 °C to obtain a black powder of iron oxide nanoparticles.

2 $${\text{FeSO}}_{4} \cdot 7{\text{H}}_{2} {\text{O}} + 2{\text{FeCl}}_{3} + 8{\text{NH}}_{4} {\text{OH}}\mathop{\longrightarrow}\limits_{{140\;^{ \circ } {\text{C}}}}^{{{\text{Hydrothermal}}}}{\text{Fe}}_{3} {\text{O}}_{4} + 6{\text{NH}}_{4} {\text{Cl}} + ({\text{NH}}_{4} )_{2} {\text{SO}}_{4} + 11{\text{H}}_{2} {\text{O}}$$

### Preparation of ZnO nanoparticles

1.83 g zinc acetate dihydrate Zn(CH_3_CO_2_)_2_·2H_2_O was dissolved in 40 mL deionized water. The mixture was dripped into NaOH (0.01 M) of pH 8.5 under stirring for 3 h. The white precipitates were obtained and aged for 24 h. The mixture was transferred to an autoclave for the hydrothermal treatment of 6 h at 160 °C. The white precipitates of ZnO NPs were washed with deionized water until pH 7 and sonicated for 30 min. The dried powder was annealed at 370 °C in a muffle furnace for 5 h to get fine ZnO NPs powder.

3 $$({\text{A}})\quad {\text{Zn}}({\text{CH}}_{3} {\text{CO}}_{2} )_{2} \cdot 2{\text{H}}_{2} {\text{O}} + {\text{NaOH}}\mathop{\longrightarrow}\limits_{{160\;^{ \circ } {\text{C}}}}^{{{\text{Hydrothermal}}}}{\text{CH}}_{3} {\text{COONa}} + {\text{Zn}}({\text{OH}}_{2} )$$

4 $$({\text{B}})\quad {\text{Zn}}({\text{OH}})2\mathop{\longrightarrow}\limits_{{370\;^{ \circ } {\text{C}}}}^{{{\text{Calcination}}}}{\text{ZnO}} + {\text{H}}_{2} {\text{O}}$$

### Fabrication of Ag@Fe_3_O_4_/ZnO nanocomposite

A precipitation method was used to fabricate the silver-doped iron zinc oxide (Ag@Fe_3_O_4_/ZnO). The 1:1 M Fe_3_O_4_ and ZnO NPs were dissolved in 30 mL ultrapure water under constant stirring for 5 h. The reaction mixture was dripped into 0.05 M NaOH solution for precipitation. The black precipitates of zinc oxide-coated iron oxide (Fe_3_O_4_/ZnO) were obtained. Then, 10 mL silver NPs were homogenized in a 30 mL solution of Fe_3_O_4_/ZnO by ultrasonication for 30 min at 60 °C. The dark red homogenous mixture of Ag@Fe_3_O_4_/ZnO was allowed to settle for 24 h and washed with deionized water and ethanol.

### Synthesis of methemoglobin from oxyhemoglobin

A 200 mL PBS buffer of pH 7.4 was used to adjust the HbFe^3+^ MetHb. To synthesize MetHb, 3.6 g standard hemoglobin (HbFe^2+^ OHb) was diluted in 200 mL PBS under constant stirring for 6 h^40^. The reactor vessel was adjusted with a suction pump to remove extra froth during the reaction, and the mixture was recirculated repeatedly. As shown in the mechanism, a 10 mL sodium nitrite (NaNO_2_) solution (0.05 g/mL) was injected through a 10 mL syringe.

**Figure 1:**
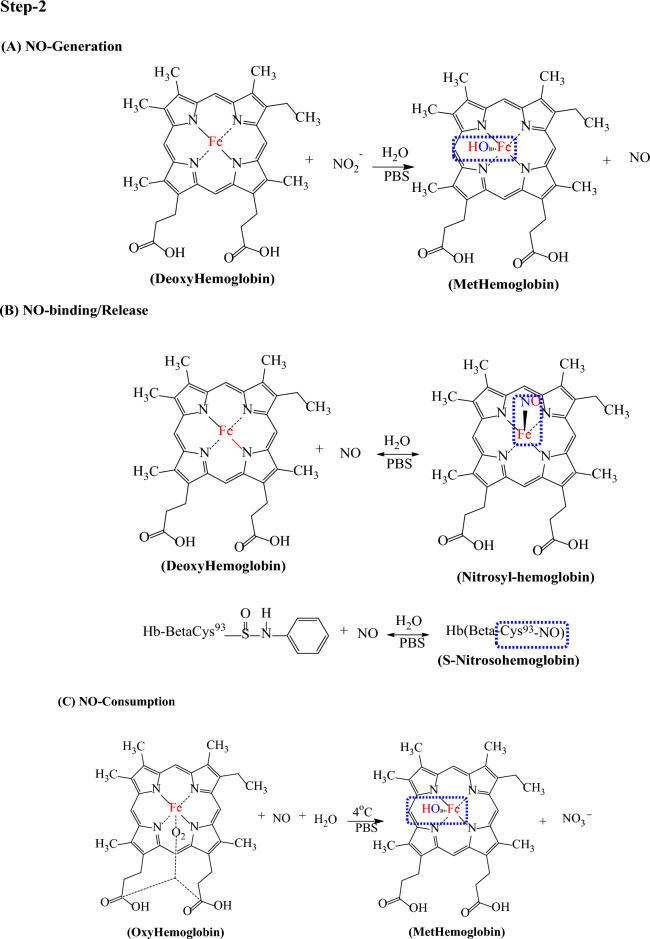
Conversion of oxyhemoglobin into methemoglobin.

After 4 h stirring and recirculating this solution, the dark brown color indicated the formation of MetHb, as shown in Fig. 1. The obtained methemoglobin solution was refrigerated at 4 °C for 1 week.

### Electrochemical sensing of methemoglobin using Ag@Fe_3_O_4_/ZnO nanocomposite

The electrical conductivity and redox reaction of Ag@Fe_3_O_4_/ZnO was evaluated by CV. A potentiostat model (CORRTEST-CS120) with Ag@Fe_3_O_4_/ZnO modified glassy carbon (GCE) as the working electrode, platinum (Pt) as a counter electrode, and Ag/AgCl as the reference electrode were used to determine the electrochemical reactions. GCE was polished with ethanol and water to avoid contamination. Ag@Fe_3_O_4_/ZnO NPs were dispersed in deionized water to obtain slurry for making reference electrodes. This slurry of Ag@Fe_3_O_4_/ZnO was deposited on GCE by micropipette and dried. HbFe^3+^ MetHb solution was diluted in different PBS concentrations. All mentioned parameters were adjusted at room temperature.

### Collection and analysis of anemic blood samples

Blood samples of anemic patients (major) were collected from Sahiwal Medical College, Sahiwal, Pakistan, with the prior approval of the Ethical Committee of Sahiwal Medical College Sahiwal Pakistan. All methods were carried out in accordance with relevant guidelines and regulations. The samples were collected from in K2-EDTA (BD-Vacutainers)^41^ with their prior informed consent of volunteers and analyzed on potentiostat to determine the comparative aspects of methemoglobin in “vivo” and modified MetHb from standard hemoglobin in “vitro”.

## Results and discussion

### Characterizations

**Figure 2:**
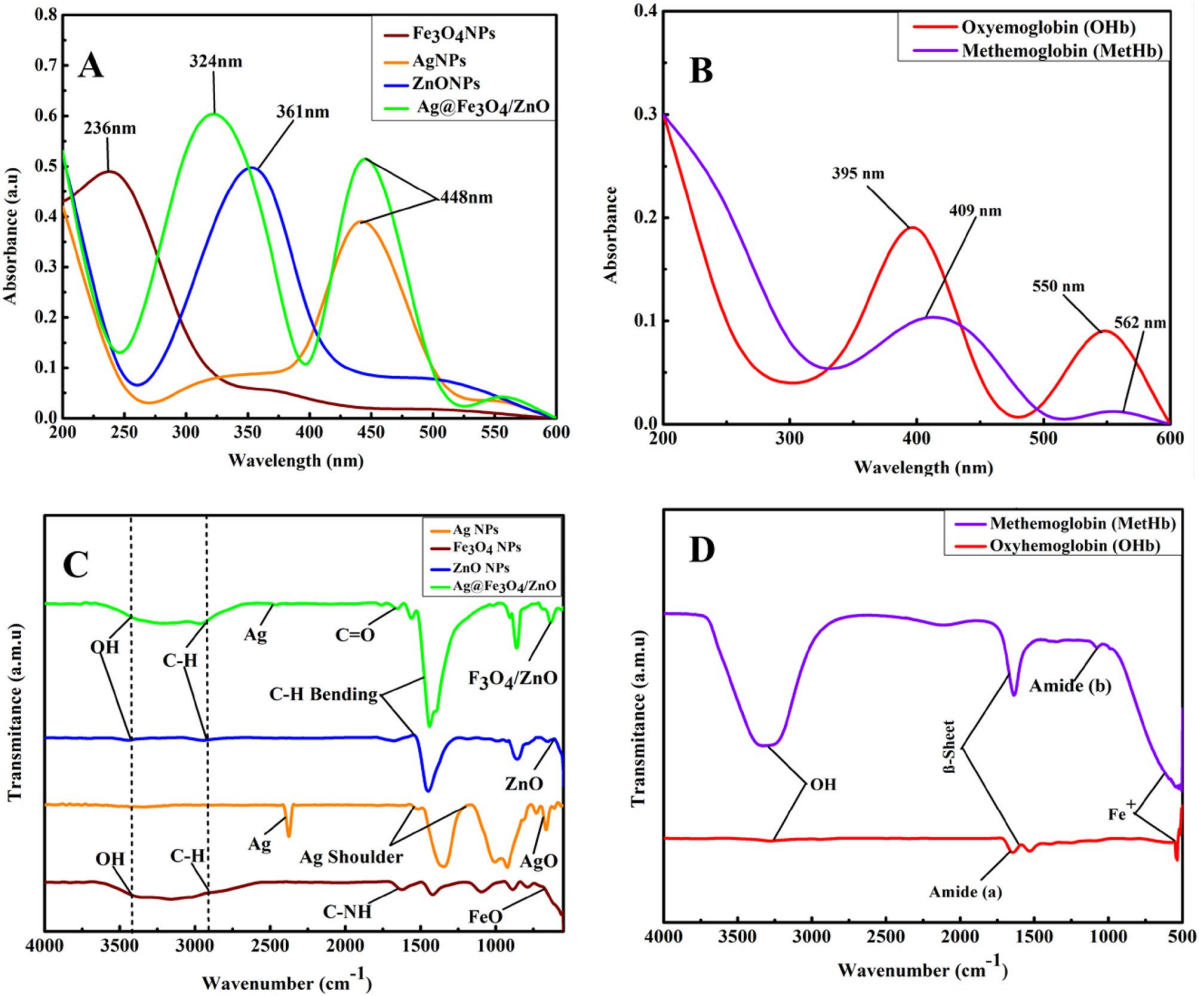
(**A**, **B**) UV and (**C**, **D**) FTIR spectra of Ag@Fe_3_O_4_/ZnO nanocomposite and MetHb/OHb, respectively.

UV–visible spectrophotometer (AQ7100APAC Thermo Fischer Scientific UK Spectrophotometer) is used to analyze Ag@Fe_3_O_4_/ZnO and MetHb/OHb at a wavelength ranging from 200 to 800 nm. UV–visible spectra of Ag@Fe_3_O_4_/ZnO and MetHb/OHb are shown in Fig. 2A,B, respectively. The absorption bands at 234 nm, 324 nm, 361 nm, and 461 nm indicate the nanocomposite formation. Figure 2B shows absorption bands at 395 nm, 409 nm, 550 nm, and 562 nm for MetHb/OHb. FTIR spectra of Ag@Fe_3_O_4_/ZnO and MetHb/OHb are shown in Fig. 2C,D*,* respectively, and are obtained by measuring transmittance from 4000 to 400 cm^−1^ on INVENIO FTIR Spectrophotometer Bruker Germany. The bands between 3500 and 2800 cm^−1^ represent OH and CH stretch. The sharp band at 2380 cm^−1^ and shoulder peak between 1600 and 1350 cm^−1^ indicate the stretching vibration of Ag NPs. The peaks from 1600 to 1700 cm^−1^ represent C=O and C–NH. The sharp peaks from 600 to 540 cm^−1^ represent pure metal and metallic oxides (FeO ZnO and Ag^+^). MetHb/OHb shows a broad band from 3300 to 3000 cm^−1^ for the hydrogen bonding of the OH bond. The amide and (α, β) sheet bands are shown at 1600 and 1100 cm^−1^. CMA in MetHb/OHb, shows a sharp band from 550 to 500 cm^−1^, as reported in the literature^42^.

**Figure 3:**
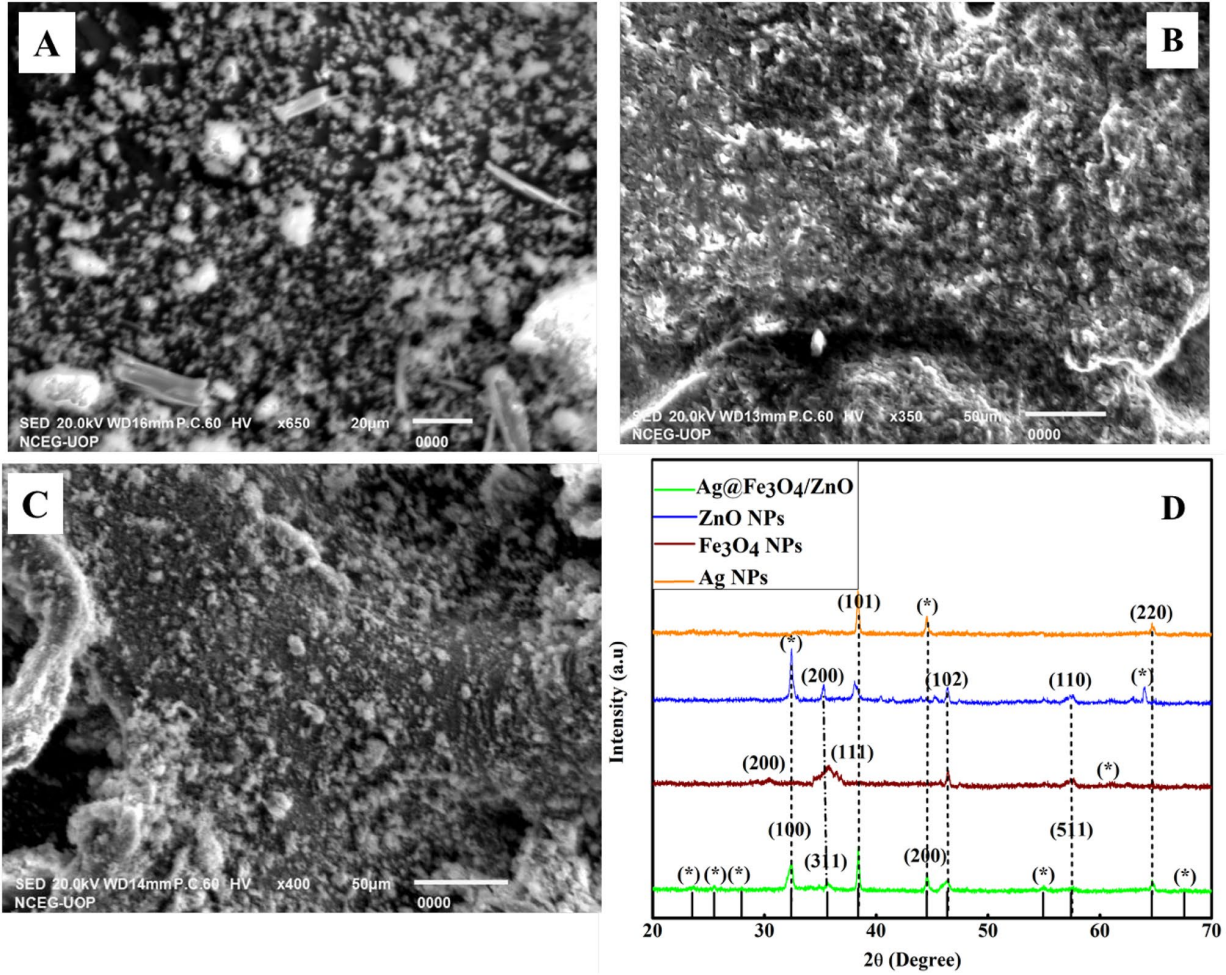
(**A**) Ag-Fe_3_O_4_, (**B**) ZnO, and (**C**) Ag@Fe_3_O_4_/ZnO represent SEM, and (**D**) depicts XRD patterns.

The prepared Ag-Fe_3_O_4_ (Fig. 3A), ZnO (Fig. 3B), and Ag@Fe_3_O_4_/ZnO (Fig. 3C) exhibit rough surfaces. X-ray diffraction (XRD) analysis data of Ag@Fe_3_O_4_/ZnO is given in Fig. 3D. The crystalline structure of Ag@Fe_3_O_4_/ZnO shows peaks at 32°, 30°, 38°, 45°, 46°, 58°, and 65° while ZnO shows at 32°, 35°, 38°, 46°, 58°, and 64°. Ag NPs show signals at 38°, 45°, and 65°, whereas Fe_3_O_4_ shows peaks at 30°, 35°, 45°, and 58°. Sharp peaks throughout the data indicate the crystallinity of Ag@Fe_3_O_4_/ZnO nanomaterial.

### Electrochemical studies

**Figure 4:**
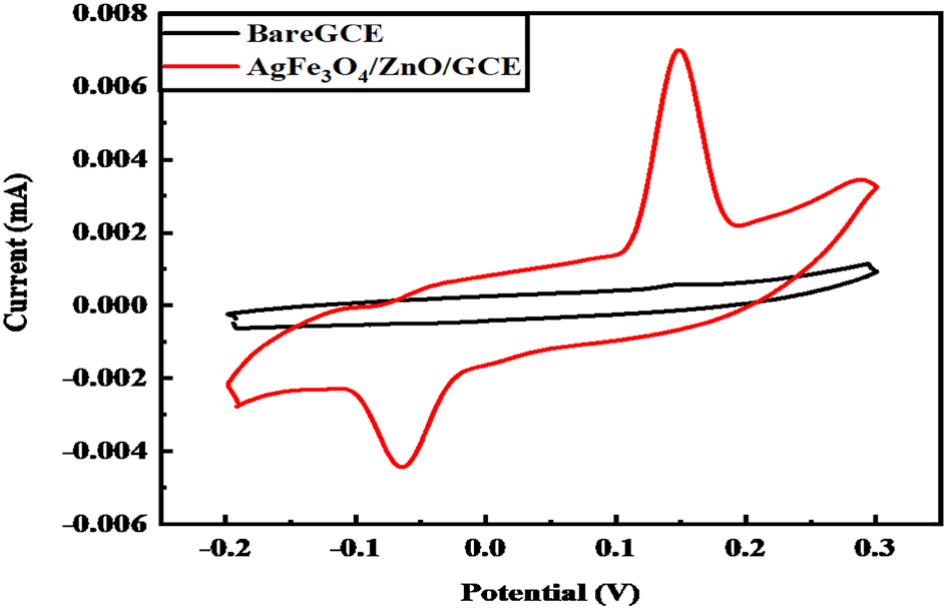
Conductance of Ag@Fe_3_O_4_/ZnO nanomaterial.

The electrochemical properties of Ag@Fe_3_O_4_/ZnO are analyzed through CV in 0.1 M K_4_[(Fe(CN_6_)]/KCl solution. The current (*I*) intensity is higher in Ag@Fe_3_O_4_/ZnO, used for the electrochemical detection of methemoglobin (MetHb) in anemic patients. Ag@Fe_3_O_4_/ZnO response toward the electrochemical signal depends on the conductivity of this nanomaterial, and the response is studied before and after the activation of the nanomaterial. The strong anodic and cathodic peaks can be seen in the voltammogram after activation, as shown in Fig. 4. The oxidation and reduction reactions of Ag@Fe_3_O_4_/ZnO modified GCE in 0.1 M K_4_[(Fe (CN_6_)]/KCl solution resulted in these prominent anodic and cathodic peaks due to efficient electron transfer. Both current signals, i.e., oxidative and reductive, become stronger after activation. Equations for oxidation (Eq. 5) and reduction (Eq. 6) are given below

5 $${\text{Ag}}@{\text{Fe}}_{{3}} {\text{O}}_{{4}} /{\text{ZnO}} + {\text{K}}_{{4}} \left[ {{\text{Fe }}\left( {{\text{CN}}_{{6}} } \right)} \right] \, \to {\text{ Ag}}@{\text{Fe}}_{{3}} {\text{O}}_{{4}} /{\text{ZnO}} + {4}\left[ {{\text{Fe }}\left( {{\text{CN}}} \right)_{{6}} } \right]^{ - } + {\text{4K}}^{ + }$$

6 $${\text{Ag}}@{\text{Fe}}_{{3}} {\text{O}}_{{4}} /{\text{ZnO}}^{ + } + {4}\left[ {{\text{Fe }}\left( {{\text{CN}}} \right)_{{6}} } \right]^{ - } + {\text{4e}}^{ - } \to {\text{ Ag}}@{\text{Fe}}_{{3}} {\text{O}}_{{4}} /{\text{ZnO}} + {\text{K}}_{{4}} \left[ {{\text{Fe }}\left( {{\text{CN}}_{{6}} } \right)} \right]$$

### Mechanism of electrochemical sensing of methemoglobin

The chemical oxidation of hemoglobin (HbFe^2+^) through (NaNO_2_) leads to the formation of methemoglobin (HbFe^3+^) in anemic patients. Electron transfer during this process determines the overall process. Ag@Fe_3_O_4_/ZnO are the electrochemical transducers for detecting methemoglobin via CV. Fe3O4/ZnO assesses the electrical conductivity and increment in surface area, and charge transfer is enhanced by Ag NPs^43^.

### Optimization of CV parameters for MetHb detection

**Figure 5:**
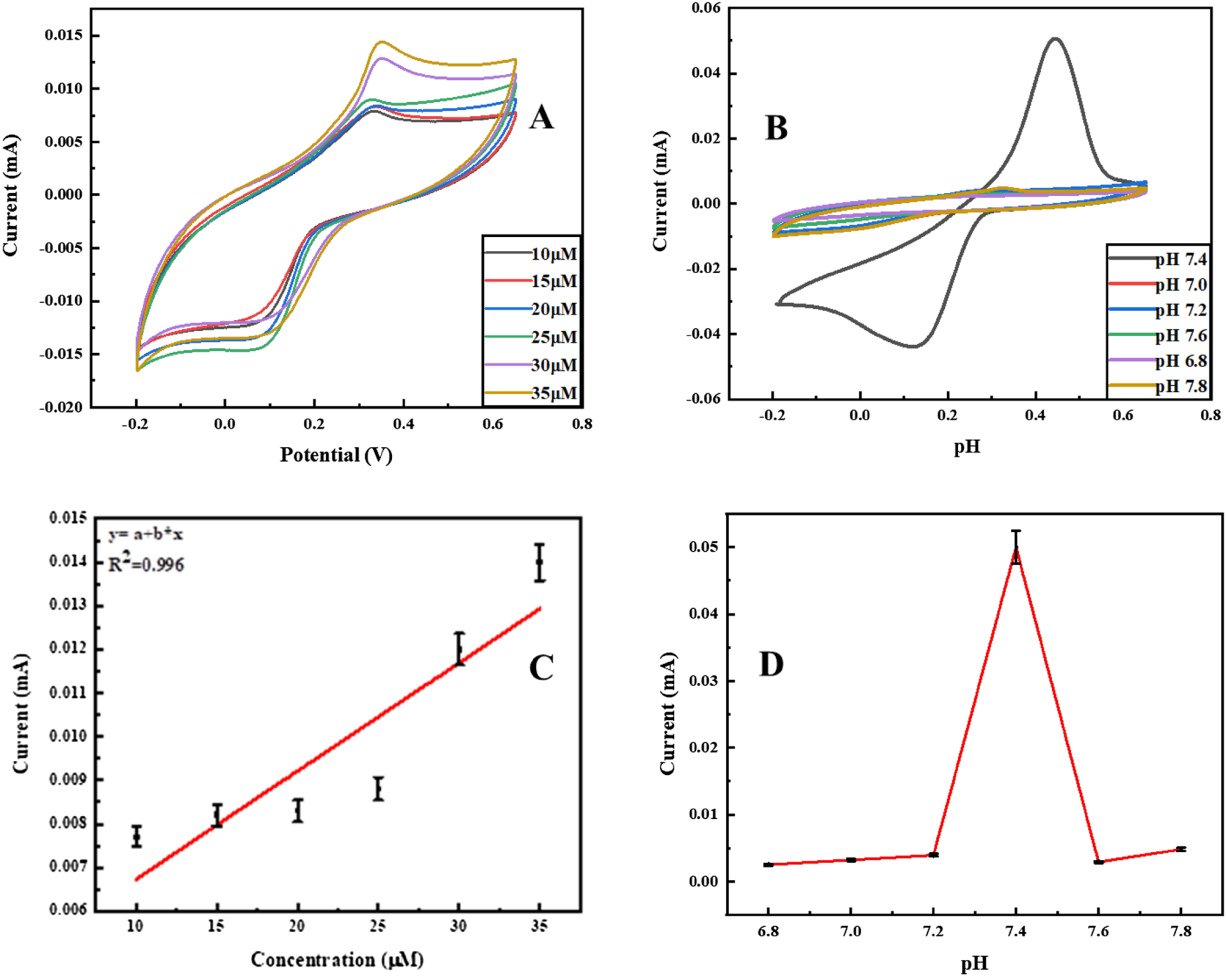
Cyclic voltammograms depicting the effects of concentration and pH (**A**, **B**) on the electrochemical behavior of Ag@Fe_3_O_4_/ZnO NPs for methemoglobin detection and their corresponding line graphs (**C**, **D**) in 0.1 M PBS.

**Figure 6:**
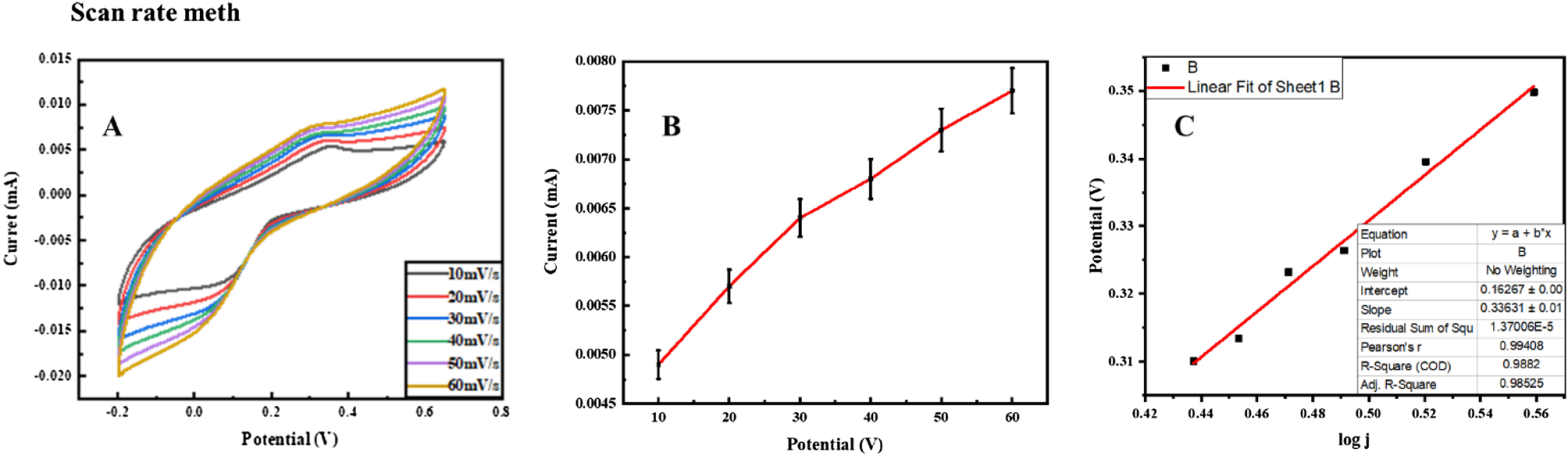
(**A**) Scan rate, (**B**) line graph for methemoglobin sensing, and (**C**) corresponding Tafel slope.

The effect of hemoglobin concentration on Ag@Fe_3_O_4_/ZnO is determined by CV (Fig. 5A). The oxidative current increases with an increase in MetHb concentration which results in the maximum oxidation signal. The maximum current is observed at 35 µM MetHb concentration, while the lowest oxidation peak current is at 10 µM in 0.1 M PBS solution of pH 7.4. The relation between current and concentration of MetHb has a correlation coefficient of R^2^ 0.996, as shown in Fig. 5C. MetHb is detected at different pH (6.8, 7.0, 7.2, 7.4, 7.6, and 7.8), and generated cyclic voltammograms are given in Fig. 5B which illustrate the maximum as well as current at different pH (Fig. 5D). Depending on the intensity and shape of MetHb signals, pH 7.4 is optimal for MetHb detection through CV in 0.1 M PBS. A significant decrease in peak current is observed at pH above or below 7.4. The effects of scan rates and fluctuations in current due to the redox behavior of Ag@Fe_3_O_4_/ZnO for MetHb sensing are shown in Fig. 6A,B.

ECSA plays an essential role in the detection of the analyte. Increased ECSA provided more reactive sites for albumin to interact with fabricated material, increasing sensor response. The sole reason is the interaction between the electrode surface and analyte generating signal, which the sensor detects.

To change is quantified via the charge transfer coefficient (α), determined by the following equation:

7 $$\upalpha = {\text{Tafel\,slope}} \times 2.303 \times \frac{{{\text{RT}}}}{{{\text{nF}}}}$$

here, R denotes the global gas constant (8.314 JK^−1^ mol^−1^), T indicates thermodynamic temperature (298.15 K), n represents the number of electrons transferred in rate determining step (i.e., 2), and F refers to Faraday constant (96,485 C mol^−1^). Thus, the obtained Tafel slope of the modified electrode is 24 mV/s (Fig. 6C). The calculated transfer coefficient is 0.71. Similarly, the obtained value of the Tafel slope for bare electrodes is 33 mV/s. The calculated transfer coefficient is 0.75. Due to increased surface area, the Tafel slope of modified GCE is lower with higher electrocatalytic activity than bare GCE since more active sites are available at modified GCE, leading to more charge transfer rate.

### Electrochemical surface area (ECSA) of Ag@Fe_3_O_4_/ZnO

ECSA of Ag@Fe_3_O_4_/ZnO is evaluated by using 0.04 mM [Fe(CN)_6_]^3−/4−^ and KCl solutions (0.1 M) in a 1:1 M ratio, respectively. To get more precise and comparable results, bare GCE and Ag@Fe_3_O_4_/ZnO NPs (SIZO-NPs) modified GCE are analyzed through the CV.

**Figure 7:**
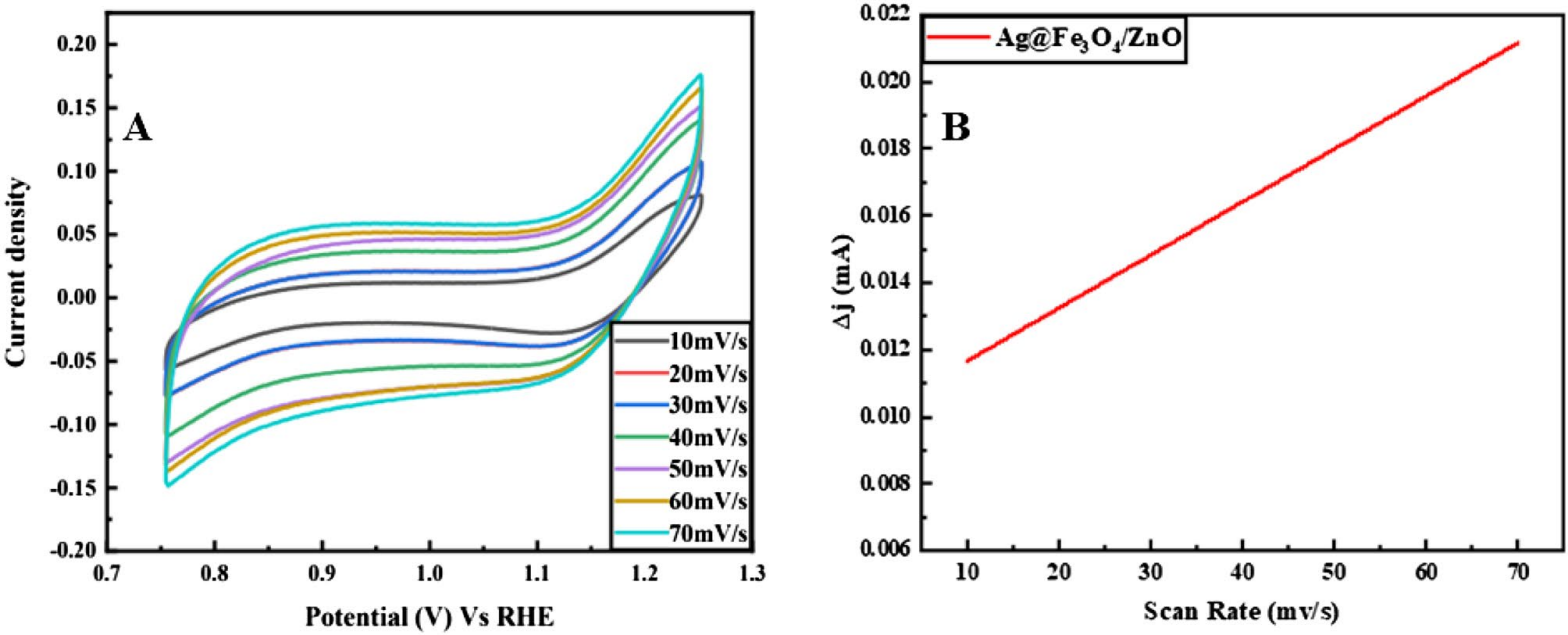
(**A**) ECSA of Ag@Fe_3_O_4_/ZnO on GCE at various scan rates, and (**B**) slope calibration of scan rates.

Both bare GCE and Ag@Fe_3_O_4_/ZnO NPs (SIZO-NPs)/GCE have shown different results. Different scan rates of Ag@Fe_3_O_4_/ZnO/GCE are taken in [Fe(CN)_6_]^3−/4−^ solution to perform the voltammetric analysis and to determine ECSA^44^. The scan rates vary between 10 to 70 mV/s in redox solution; the outcome is shown in Fig. 7A,B. The obtained surface area of Ag@Fe_3_O_4_/ZnO modified GCE is 0.0791 cm^2^, and for the unmodified GCE is 0.073 cm^2^.

Whereas the kinetic parameter is calculated from the following equation:

$$\Psi = (0.6288 + 0.021{\text{X}})/(1 - 0.017{\text{X}})$$

X is the ΔEP is used to determine ψ as a function of ΔE_P_ from the experimentally recorded voltammetry. Thus, the obtained value of the kinetic parameter (ψ) is 0.63.

### The catalytic reaction rate constant (kcat)

The value of the catalytic reaction rate constant (kcat) is determined by the following equation:

$$\text{k}^\circ =\frac{\text{RT}}{{\text{F}}^{2}{\text{R}}_{\text{act}}\text{AC}}$$

here, R, T, F, R_act_, A, and C represents the global gas constant (R = 8.314 JK^−1^ mol^−1^), thermodynamic temperature (T = 298.15 K), faraday constant (F = 96,485 C mol^−1^), charge transfer resistance (R_ct_ = 35,867 Ω cm^2^), electrode surface area (A = 0.07), the concentration of the electrolyte in 0.1 KOH (C), respectively. The catalytic reaction rate constant (kcat) is 1.06 × 10^–8^.

### Transfer coefficient (α)

The transfer coefficient is determined by employing the following equation:

$${\upalpha }=\text{Tafel slope}\times 2.303\times \frac{{\rm RT}}{{\rm nF}}$$

here, R denotes the global gas constant (8.314 JK^−1^ mol^−1^), T denotes thermodynamic temperature (298.15 K), n denotes the number of electrons transferred in the rate-determining step, i.e., 2 and F refer to the faraday constant (96,485 C mol^−1^). The calculated transfer coefficient is found to be 0.71.

### Tafel slope (b)

Tafel slope (b) can be determined by employing the following equation:

$$\text{Y}=\text{a}+{\text{b}}^{2}\text{X}$$

**Figure 8:**
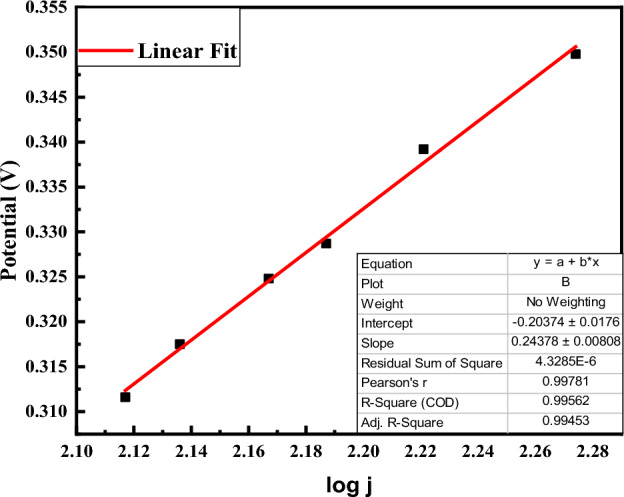
Tafel slope of the Ag@Fe_3_O_4_/ZnO modified GCE for methemoglobin sensing.

The calculated Tafel slope (b) value is 0.24 mV/dec (Fig. 8).

### Selectivity factor

**Figure 9:**
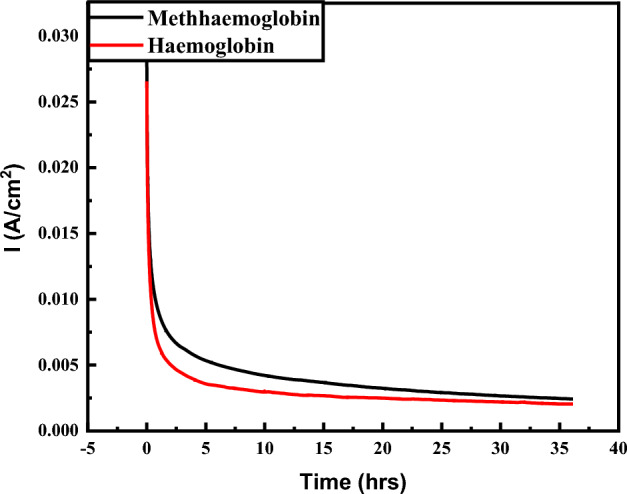
Selectivity factor of the Ag@Fe_3_O_4_/ZnO modified GCE for methemoglobin sensing.

The selectivity factor of the sensor is calculated by using the following equation:

$${\text{i}}_{{\text{t}}} = {\text{K}}({\text{C}}_{{\text{i}}} + \Sigma {\text{k}}_{{{\text{ij}}}}^{{{\text{amp}}}} {\text{C}}_{{\text{j}}} )$$

where Ci, Cj, i_t,_ and K are the concentrations of the target analyte, the concentration of interfering species at 35 µM, total current response, and catalytic reaction rate constant, i.e., 1.06 **× **10^–8^, respectively. Σk_ij_^amp^ is the amperometric selectivity coefficient, a measure of the preference of the sensor for the analyte relative to the interferents (Fig. 9).

Σk_ij_^amp^ is found to be 3.49.

### Roughness factor (fr)

The fr is the ratio between peak current I_p2_ and surface area A_2_ of material (Ag@Fe_3_O_4_/ZnO) to the peak current I_p1_ and surface area A_1_ of blank GCE as shown in Eq. (8). The given formula calculates the roughness factor:

8 $$fr=\frac{Ip2}{Ip1}=\frac{A2}{A1}$$

The electrode dimensions and quantity of redox centers on the analyte surface determine the roughness factor's strength (fr). It is intended by I_pa_ of ferrocyanide [Fe(CN)_6_]^3−/4−^ through redox couple equaling to blank GCE. The electrode surface area ratio is equivalent to the oxidation ratio between two electrodes, representing the change in actual surface area. The actual dimensions of the electrode followed by an electrochemical pathway and redox cores existing on the surface are responsible for the fluctuation in fr^45^. The surface areas of Ag@Fe_3_O_4_/ZnO (A_2_) and blank GCE (A_1_) are 0.0791 cm^2^ and 0.073 cm^2^, respectively, and the calculated fr is 1.08.

### Stability of Ag@Fe_3_O_4_/ZnO/GCE

**Figure 10:**
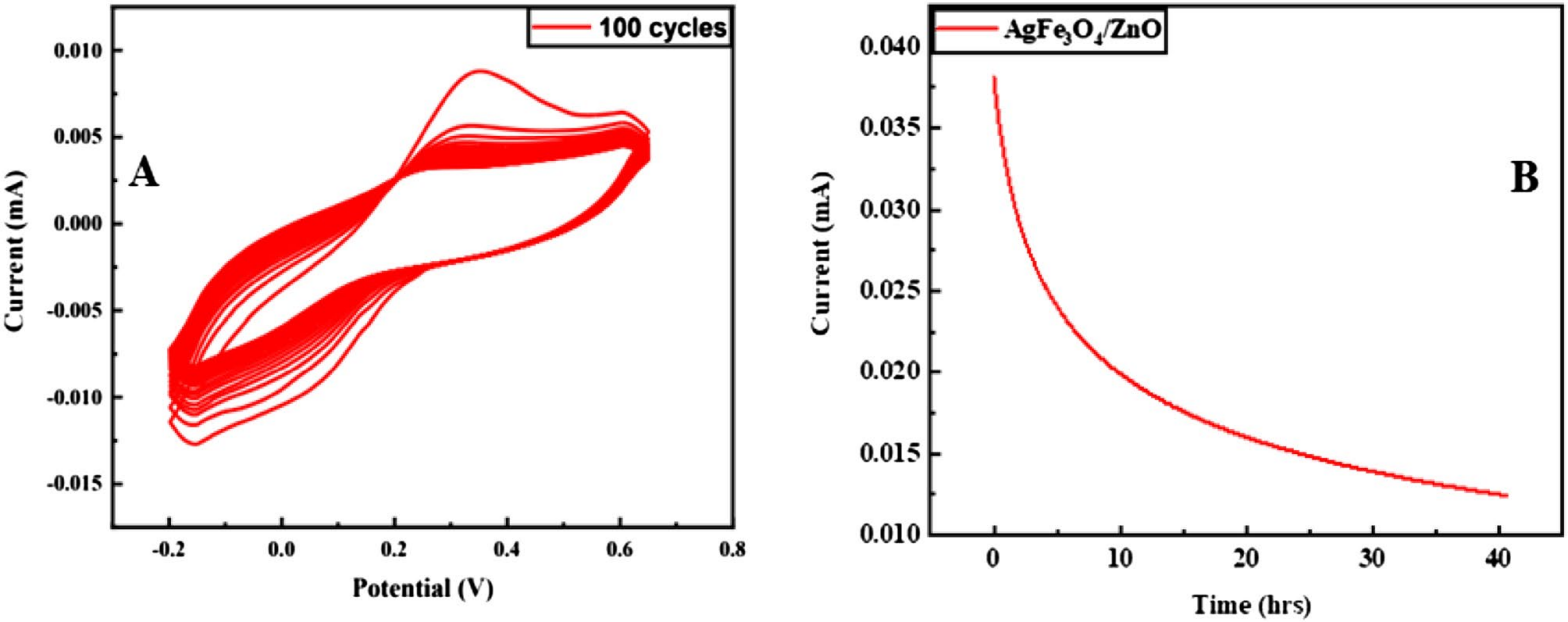
(**A**) Stability of Ag@Fe_3_O_4_/ZnO at 100 cycles, and (**B**) chronoamperometry of CV.

The stability of modified electrode Ag@Fe_3_O_4_/ZnO/GCE is determined by running 100 cycles with 1 µM of modified hemoglobin, i.e., methemoglobin (MetHb) in 0.1 M PBS of pH 7.4, as shown in Fig. 10A. After 100 cycles, the redox signal is measured at the same potential as in the first cycle. Ag@Fe_3_O_4_/ZnO/GCE can reproducibly be used many times. The further steady-state durability of Ag@Fe_3_O_4_/ZnO is determined by chronoamperometry, as shown in Fig. 10B at 0.038 V for 40 h with suitable concentrations.

### Electrochemical impedance spectroscopy (EIS) of Ag@Fe_3_O_4_/ZnO

**Figure 11:**
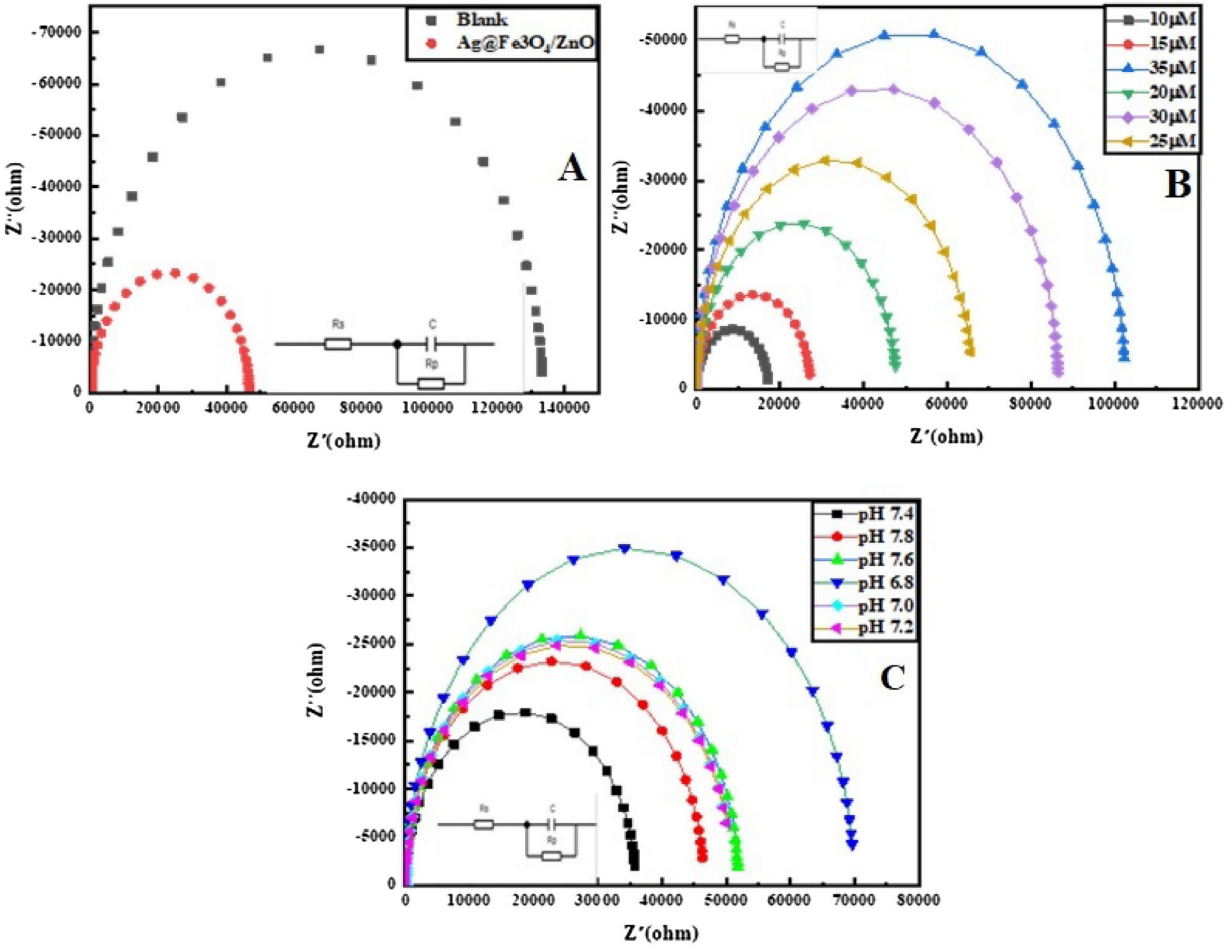
(**A**) Impedance graphs of unmodified GCE and Ag@Fe_3_O_4_/ZnO modified GCE in [Fe(CN)_6_]^3−/4−^ and KCl (**B**) at various concentrations of methemoglobin, and (**C**) at different pH.

EIS assesses the charge transfer mechanism of sensing material and stepwise modification. In Fig. 11A, impedance is measured on Ag@Fe_3_O_4_/ZnO using 0.1 M K_3_[Fe(CN)_6_] and 0.1 M KCl. The concentration effect of methemoglobin (HbFe^+3^) on impedance from 10 to 35 µM is given in Fig. 11B. The interfacial charge transfer increases with an increase in the concentration of MetHb, and results depict that electrostatic forces of attraction are related to the concentration of MetHb and produce alterations in charge transfer resistance of MetHb on the modified electrode surface.

MetHb molecules are bulky, which causes the steric hindrance toward charge transfer. The effect of pH is determined by applying impedance at sequential pH of 6.8. 7.0, 7.2, 7.4, 7.6, and 7.8, as shown in Fig. 11C*.* In the equivalent circuit diagram, R_p_ is the electron transfer resistance on the surface of an electrochemical sensor, R_s_ is solution resistance, and C_dl_ is the component capacitance of the electrochemical sensor.

### Electron transfer rate constant (k°) in heterogeneous phase

The electrochemical cells work in an electro-catalytic solution under alternating current (AC) potential. Hence, EIS determines the charge distribution on electrodes by applying sinusoidal perturbation in continuous linear and semicircular segment circuits. The interfacial capacitance (CdI), Ohmic resistance (Rs), electron transfer resistance (Rct), and Warburg impedance (Zw) are the major components of a continuous circuit. The electron transfer resistance (Rct) detects electron transference in a redox reaction and is measured by semicircular diameter. Ag@Fe_3_O_4_/ZnO NPs enhance the electron transfer rate between electrodes due to excess conductivity and reliability. Linearity in signal is due to dispersion at lower frequencies. GCE's electron transfer resistance (Rct) is ~ 27,249 Ω, and Ag@Fe_3_O_4_/ZnO is 17,172 Ω, respectively. The relationship is determined between surface resistance and electrical conductivity of Ag@Fe_3_O_4_/ZnO.

The conductivity of Ag@Fe_3_O_4_/ZnO NPs is greater compared to bare GCE. The minor difference in conductivity of nanomaterial and GCE is responsible for the change in reduction potential as Ag@Fe_3_O_4_/ZnO has a large surface area and higher electrical conductivity. They imply an impact on the selectivity and sensitivity of EIS.

9 $$\text{k}^\circ =\frac{\text{RT}}{{F}^{2}\text{RctAC}}$$

k° is determined by the above equation, where F is Faraday constant (96,485 C mol^−1^), Rct is electron transfer resistance (27,249 Ω for GCE, and 17,172 Ω for Ag@Fe_3_O_4_/ZnO/GCE), T is the thermodynamic temperature (~ 298.15 K), R is the global gas constant (8.314 J K^−1^ mol^−1^), A is electrode surface area (0.073 cm^2^ for bare GCE and 0.0791 cm^2^ for Ag@Fe_3_O_4_/ZnO/GCE), C is [Fe(CN)_6_]^3−/4−^ solution concentration (0.1 mM cm^−3^), and k° is the rate constant of standard heterogeneous electron transfer (cm s^−1^). k° for Ag@Fe_3_O_4_/ZnO/GCE and for unmodified GCE are 1.96 × 10^–10^ cm s^−1^ and 1.3 × 10^−10^ cm s^−1^, respectively. k° represents the approximate kinetics of redox pairs, and the system having higher k° establishes equilibrium in less time, depicting a faster electron transfer rate.

### Determination of limit of detection (LOD)

The alternative, dependent derivative, i.e., the limit of detection (LOD), determines the kinetics and completion of the chemical reaction with actual concentration. LOD refers to the minimum concentration of the analytical sample, which can be distinguished by zero in an analyte. LOD varies with the influence of reaction conditions and the redox pH of analytical components. The following equations can determine LOD:

10 $$\text{LOD}=3\frac{{\rm s}}{{\rm m}}$$

here “s” represents the standard deviation, “m” is the slope, and the calculated LOD is 0.17 µM.

### MetHb detection in blood serum and recovery analysis

**Figure 12:**
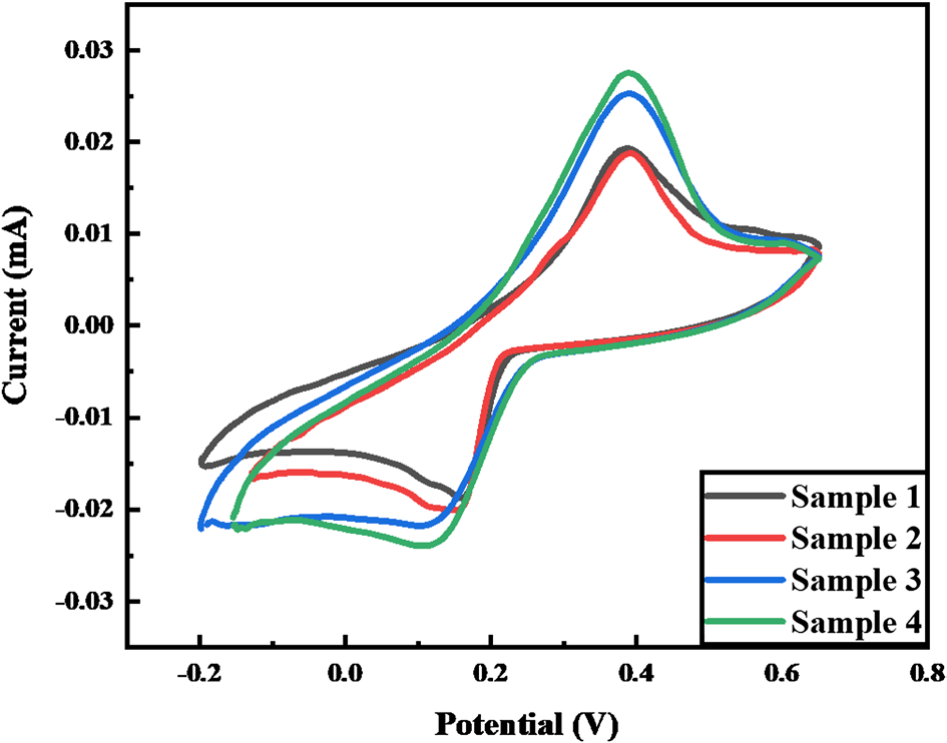
Cyclic voltammogram showing the detection of methemoglobin in the serum of anemic patients.

Ag@Fe_3_O_4_/ZnO NPs show peculiar characteristics as electro-catalyst. The oxidized form of “heme” (Fe^3+^) in serum samples of methemoglobinemia patients is analyzed by a CV to verify the applicability of the modified Ag@Fe_3_O_4_/ZnO sensor. Blood samples (S1, S2, S3, and S4) are collected from anemic patients. The samples are diluted in a buffer of pH 7.4, and the recovery spike is denoted over the oxidative and reductive curves of cyclic voltammograms, as shown in Fig. 12.

Samples show a maximum oxidation form of standard hemoglobin in anemic patients, which refers to anemia. The recovery percentage ranges from 88.8 to 97.7% of MetHb, as shown in Table 1. Oxidation and reduction signals accurately detect methemoglobin levels in anemic patients.

**Table 1:** Hemoglobin recovery studies from serum samples.

Sample	Added concentration (µM)	Calculated concentration (µM)	Recovery (%)
S1	35	31.1	88.8
S2	35	32.1	91.7
S3	35	33.8	96.5
S4	35	34.2	97.7

## Conclusions

An electrochemical homogeneous deposition route has been adopted for sensing methemoglobin (HbFe^3+^) in anemic patients. The redox reaction in cyclic voltammetric (CV) setup verifies the sensing of a biological analyte. All the parameters of CV, i.e., electrical conductivity, stability, selectivity chronoamperometry, pH, concentration, and ECSA, are optimized in phosphate buffer (PBS) and potassium ferrocyanide [Fe(CN)_6_]^3−/4−^ solutions. The hydrothermal methodology has been adopted for synthesizing Ag@Fe_3_O_4_/ZnO electrocatalyst. The redox method is used for preparing methemoglobin from the standard form of oxyhemoglobin. The as-synthesized Ag@Fe_3_O_4_/ZnO sensor induces biosensing of methemoglobin oxidation and reduction with high sensitivity of 0.17 µM HbFe^3+^ with fast response, selectivity, and stability in 0.1 M PBS. Results reveal the potential of Ag@Fe_3_O_4_/ZnO NPs as a biosensor for application in clinical trials of detecting methemoglobin.

## Data Availability

All data generated or analysed during this study are included in this published article.

## References

[1] CHAIN, N.O.V.B.G. (2020). Rodak's hematology clinical principles and applications. Platelets.

[2] Rangan A (2021). Interpreting sulfhemoglobin and methemoglobin in patients with cyanosis: An overview of patients with M-hemoglobin variants. Int. J. Lab. Hematol..

[3] Asnaashari M (2019). An electrochemical biosensor based on hemoglobin-oligonucleotides-modified electrode for detection of acrylamide in potato fries. Food Chem..

[4] Hussain KK (2017). Electrochemical detection of hemoglobin: A review. Electroanalysis.

[5] Ahmed MH, Ghatge MS, Safo MS (2020). Hemoglobin: Structure, function and allostery. Vertebr. Invertebr. Respirat. Proteins Lipoproteins Other Body Fluid Proteins.

[6] Eissa S, Zourob M (2017). Aptamer-based label-free electrochemical biosensor array for the detection of total and glycated hemoglobin in human whole blood. Sci. Rep..

[7] Melkani DC (2020). Study of proteomic diversity for sickle cell disease in tribe and non-tribe population of Kumaun Region of Uttarakhand. Bull. Environ. Pharmacol. Life Sci..

[8] Gupta A (2020). Biochemical Parameters and the Nutritional Status of Children: Novel Tools for Assessment.

[9] Kiese M (2019). Methemoglobinemia: A Comprehensive Treatise: Causes, Consequences, and Correction of Increased Contents of Ferrihemoglobin in Blood.

[10] Posta N (2020). Hemoglobin oxidation generates globin-derived peptides in atherosclerotic lesions and intraventricular hemorrhage of the brain, provoking endothelial dysfunction. Lab. Invest..

[11] Kashari OF (2022). Occurrence of methemoglobinemia due to COVID-19: A case report. Cureus..

[12] Ara T, Haque QS, Afrose S (2019). A rare case of congenital methemoglobinemia with secondary polycythemia-case report and literature review. Haematol. J. Bangladesh.

[13] Soliman DS, Yassin M (2018). Congenital methemoglobinemia misdiagnosed as polycythemia vera: Case report and review of literature. Hematol. Rep..

[14] Iolascon A (2021). Recommendations for diagnosis and treatment of methemoglobinemia. Am. J. Hematol..

[15] Achille, I., *et al*. *Recommendations for diagnosis and treatment of methemoglobinemia.* 10.1002/ajh.26340 PMC9291883 34467556

[16] Jensen B, Fago A (2018). Reactions of ferric hemoglobin and myoglobin with hydrogen sulfide under physiological conditions. J. Inorg. Biochem..

[17] Keszler A (2008). The reaction between nitrite and oxyhemoglobin: A mechanistic study. J. Biol. Chem..

[18] Pourreza N, Golmohammadi H (2015). Hemoglobin detection using curcumin nanoparticles as a colorimetric chemosensor. RSC Adv..

[19] Myrgorodska I (2016). Enantioselective gas chromatography in search of the origin of biomolecular asymmetry in outer space. Isr. J. Chem..

[20] Zhou Y (2015). Fabrication of electrochemical interface based on boronic acid-modified pyrroloquinoline quinine/reduced graphene oxide composites for voltammetric determination of glycated hemoglobin. Biosens. Bioelectron..

[21] Li J (2020). Recent progress in flexible and stretchable piezoresistive sensors and their applications. J. Electrochem. Soc..

[22] Fatima B (2020). Tellurium doped zinc imidazole framework (Te@ ZIF-8) for quantitative determination of hydrogen peroxide from serum of pancreatic cancer patients. Sci. Rep..

[23] Naqvi STR (2020). Fabrication of iron modified screen printed carbon electrode for sensing of amino acids. Polyhedron.

[24] Klimuntowski M (2020). Electrochemical sensing of cannabinoids in biofluids: A noninvasive tool for drug detection. ACS Sensors.

[25] Gao Y (2020). Flexible hybrid sensors for health monitoring: Materials and mechanisms to render wearability. Adv. Mater..

[26] Chung D, Gray B (2019). Development of screen-printed flexible multi-level microfluidic devices with integrated conductive nanocomposite polymer electrodes on textiles. J. Electrochem. Soc..

[27] Jain U, Chauhan N (2017). Glycated hemoglobin detection with electrochemical sensing amplified by gold nanoparticles embedded N-doped graphene nanosheet. Biosens. Bioelectron..

[28] Fini H, Kerman K (2020). Revisiting the nitrite reductase activity of hemoglobin with differential pulse voltammetry. Anal. Chim. Acta.

[29] Tom J, Andreas HA (2017). The influence of carbon-oxygen surface functional groups of carbon electrodes on the electrochemical reduction of hemoglobin. Carbon.

[30] Sun AC, Hall DA (2019). Point-of-care smartphone-based electrochemical biosensing. Electroanalysis.

[31] Kalambate PK (2019). Recent advances in MXene-based electrochemical sensors and biosensors. TrAC, Trends Anal. Chem..

[32] Lete C (2020). Nitrite electrochemical sensing platform based on tin oxide films. Sens. Actuators B Chem..

[33] Rajput JK (2020). Alkali metal (Na/K) doped graphitic carbon nitride (g-C3N4) for highly selective and sensitive electrochemical sensing of nitrite in water and food samples. J. Electroanal. Chem..

[34] Shi F (2022). Pt-doped FeP-C hollow nanorod and hemoglobin based electrochemical biosensor and its applications. Int. J. Electrochem. Sci.

[35] Rafiq HS (2021). Selective electrochemical sensing of hemoglobin from blood of β-thalassemia major patients by tellurium nanowires-graphene oxide modified electrode. Chem. Eng. J..

[36] Amini N, Maleki A (2020). Electrochemical behavior of ticlopidine and detection of ethanol based on Hemoglobin/Ticlopidine/Titanium oxide NPs nanobiocomposite modified electrode. J. Electroanal. Chem..

[37] Chen W (2018). Boron-doped graphene quantum dots modified electrode for electrochemistry and electrocatalysis of hemoglobin. J. Electroanal. Chem..

[38] Canfarotta F, Rapini R, Piletsky S (2018). Recent advances in electrochemical sensors based on chiral and nano-sized imprinted polymers. Curr. Opin. Electrochem..

[39] Guzmán MG, Dille J, Godet S (2009). Synthesis of silver nanoparticles by chemical reduction method and their antibacterial activity. Int. J. Chem. Biomol. Eng..

[40] Gu X (2022). Scalable manufacturing platform for the production of methemoglobin as a non-oxygen carrying control material in studies of cell-free hemoglobin solutions. PLoS ONE.

[41] Mehmood R (2018). Evaluation of di-potassium and tri-potassium EDTA evacuated tubes for routine haematological testing. J. Clin. Lab. Anal..

[42] Saeed A, Abolaban F (2021). Spectroscopic study of the effect of low dose fast neutrons on the hemoglobin structure. Spectrochim. Acta Part A Mol. Biomol. Spectrosc..

[43] Özcan A, Hamid F, Özcan AA (2021). Synthesizing of a nanocomposite based on the formation of silver nanoparticles on fumed silica to develop an electrochemical sensor for carbendazim detection. Talanta.

[44] Sanko V (2022). An electrochemical sensor for detection of trace-level endocrine disruptor bisphenol A using Mo2Ti2AlC3 MAX phase/MWCNT composite modified electrode. Environ. Res..

[45] Kubarkov AV (2021). Electrochemical synthesis of 3D microstructured composite films of poly (3, 4-ethylenedioxythiophene) and reduced nanographene oxide. Electrochim. Acta.

